# Accuracy of Truenat™ MTB Plus for the diagnosis of pulmonary TB

**DOI:** 10.5588/ijtldopen.24.0561

**Published:** 2025-04-09

**Authors:** M.C.M. Melo, A.K. Silveira, A.P.R. Dalvi, J. Silva, V.S. Printes, L. Anselmo, T.I. da Silva, M.M. Oliveira, C. Conceição, A. Lopes, V. Bollela, M. Cordeiro-Santos, M. Bhering, A.S.R. Moreira, A.C.C. Carvalho, A.L. Kritski

**Affiliations:** ^1^ Institute of Thoracic Diseases, Medical School, Federal University of Rio de Janeiro, Rio de Janeiro, RJ, Brazil;; ^2^ Molecular Mycobacteriology Laboratory, Federal University of Rio de Janeiro, Rio de Janeiro, RJ, Brazil;; ^3^ Mycobacteriology Laboratory, Tropical Medicine Foundation Doctor Heitor Vieira Dourado, Manaus, DF, Brazil;; ^4^ Ribeirão Preto School of Medicine, University of São Paulo, Ribeirão Preto, SP, Brazil;; ^5^ Medical School, State University of Amazonas, Manaus, DF, Brazil;; ^6^ Medical School, Nilton Lins University, Manaus, DF, Brazil;; ^7^ National School of Public Health Sergio Arouca, Fiocruz, Rio de Janeiro, RJ, Brazil;; ^8^ Laboratory of Innovation, Therapies, Teaching and Bioproducts, Oswaldo Cruz Institute, Fiocruz, Rio de Janeiro, RJ, Brazil.

**Keywords:** drug-resistant tuberculosis, molecular diagnostic techniques, sensitivity and specificity, diagnosis, *Mycobacterium tuberculosis*

## Abstract

**BACKGROUND:**

Truenat™ is a WHO-recommended rapid molecular test for diagnosing TB and detecting rifampicin resistance, whose performance has been evaluated in a few high TB burden countries.

**METHODS:**

A prospective multicentre study was conducted in Brazil to estimate the sensitivity and specificity of Truenat MTB Plus compared to Xpert^®^ MTB/RIF Ultra for detecting pulmonary TB. Liquid culture for *Mycobacterium tuberculosis* and drug susceptibility testing were used as reference.

**RESULTS:**

Among 283 participants, 112 (39.6%) had culture-positive pulmonary TB. The sensitivity and specificity of Truenat MTB Plus were respectively 72.7% (95% CI 63.41–80.78) and 99.4% (95% CI 96.71–99.98), compared to 78.4% (95% CI 69.56–85.63) and 99.4% (95% CI 96.67–99.98) for Xpert MTB/RIF Ultra. In 89 people living with HIV (PLHIV), Truenat MTB Plus showed a sensitivity of 45.0% (95% CI 23.06–68.47) and specificity of 100.0% (95% CI 95.64–100.00). Among 71 patients previously treated for TB, Truenat MTB Plus showed sensitivity and specificity of respectively 79.3% (95% CI 60.28–92.01) and 97.5% (95% CI 86.84–99.94). Xpert MTB/RIF Ultra detected rifampicin resistance in 11/88 samples (12.5%) vs 9/72 (12.5%) with Truenat MTB Plus.

**CONCLUSION:**

The Truenat MTB Plus performance was comparable to Xpert MTB/RIF Ultra. Both tests demonstrated lower sensitivity in PLHIV.

The WHO recommends nucleic acid amplification tests (NAATs) as the initial diagnostic method to reduce the gap between drug-susceptible (DS) and drug-resistant (DR) TB.^1–3^ Until 2019, the WHO recommended Xpert^®^ MTB/RIF Ultra (Cepheid, Sunnyvale, CA, USA) or MTDBDR*plus* (Hain Lifesciences, Nehren, Germany), a line-probe assay (LPA), as the initial NAATs for detecting pulmonary TB, both DS and DR.^4^ Globally, NAATs increased bacteriologically confirmed TB (40–55%) and reduced the time from screening to treatment initiation (from 14 days to less than 1 week). However, NAATs did not reduce the time between symptom onset and the diagnosis, the proportion of reported cases, or TB mortality.^5–7^ Despite the incorporation of molecular tests, only 48% of bacteriologically confirmed TB cases were tested for rifampicin (RIF) resistance in 2023,^3^ highlighting persistent diagnostic gaps. In Brazil, 80,012 new TB cases were reported in 2023. Xpert MTB/RIF Ultra and culture were conducted among untreated patients in only 51.2% and 28.2% of cases, respectively. For previously treated patients, phenotypic drug susceptibility testing (DST) was performed in 51.5% of cases.^8^ New diagnostic strategies are urgently needed for rapid TB diagnosis and appropriate treatment in high-burden countries like Brazil.^9^ In 2020, the WHO recommended the use of Truenat™ (MTB or MTB Plus assay; Molbio Diagnostics, Verna, India), a chip-based real-time micro-polymerase chain reaction (PCR) for detecting *Mycobacterium tuberculosis* (MTB) DNA and RIF resistance directly from sputum. Truenat is portable, can be used in low-resource labs, runs on battery power for up to 8 h, and provides results in less than 1 h, making it applicable in areas with unreliable electricity. It also functions in humid, dusty settings with temperatures up to 40°C.^10^ However, few studies have assessed Truenat MTB Plus sensitivity and specificity in high-burden countries,^11–18^ and even fewer have compared it with Xpert MTB/RIF Ultra in patients with presumptive pulmonary TB (pPTB).^11,18^ The WHO emphasises the need for further studies to evaluate the accuracy of the new NAATs across various populations, including people living with HIV (PLHIV), patients with a history of TB, and those with pulmonary and extrapulmonary TB, both in adults and children.^1^ This study presents the accuracy results of Truenat MTB Plus in patients with pPTB from four high TB burden Brazilian cities.

## METHODS

### Study design

A prospective multicenter study evaluated the performance of the Truenat MTB Plus test and RIF assay in respiratory samples from individuals with pPTB. Liquid culture using Mycobacteria Growth Indicator Tube (MGIT; BD, Franklin Lakes, NJ, USA) was the golden standard for TB diagnosis, and MGIT SIRE was the reference for detecting RIF resistance.

### Study sites

The study was conducted in four TB clinics in Brazil: TB Outpatient Clinic at the Federal University of Rio de Janeiro (Rio de Janeiro, RJ, Brazil), Municipal Center of Duque de Caxias (Duque de Caxias, RJ, Brazil), Faculty of Medicine of Ribeirão Preto (Ribeirão Preto, SP, Brazil), and Tropical Medicine Foundation Doctor Heitor Vieira Dourado (Manaus, DF, Brazil).

### Participant eligibility and inclusion criteria

Individuals with pPTB attending the sites between July 2021 and December 2023 were consecutively screened and enrolled. Eligible participants were aged ≥18 years, had cough lasting 2 weeks or more, and at least one other TB symptom (fever, weight loss, night sweats or haemoptysis). They were required to provide sputum samples at recruitment, and 20% of participants initially classified as non-TB were asked to return for a follow-up visit approximately 2 months later if TB diagnosis was not confirmed at baseline by culture or Xpert results. Those with prior TB treatment within 60 days of enrolment were excluded.

### Definitions

Active PTB was confirmed using MGIT as the gold standard test for diagnosis in at least one sputum sample. Patients with pPTB who had negative cultures for MTB on respiratory specimens were classified as patients without TB.

MGIT results were categorised following FIND (Foundation for Innovative New Diagnostics) recommendations:^19^ if either Day 1 or Day 2 MGIT results were positive, the culture for MTB was considered positive. If both results were negative, or one was negative and the other was inconclusive or missing, the culture for MTB was considered negative. If both results were inconclusive or contaminated, the results were deemed missing. Xpert MTB/RIF Ultra results were classified as positive for MTB detection, negative for MTB undetected, and inconclusive for indeterminate results. Similarly, Truenat MTB Plus results were reported as MTB detected, MTB undetected, or indeterminate. When we started the project, the Truenat equipment did not identify low and very low samples. We did not collect information about low or very low Xpert results. Thus, Xpert and Truenat results were not classified by bacillary load but reported as MTB detection present or not. Trace results on Xpert MTB/RIF Ultra were considered positive only in individuals with HIV infection who had an MGIT-positive result.

### Sample collection

After written informed consent was obtained, two sputum samples (S1, S2) were collected from participants at recruitment, who were then instructed to collect a third sample (S3) at home the next day and return to the clinic, where a fourth sample (S4) was collected.

To compare Truenat MTB Plus with Xpert MTB/RIF Ultra, specimens from participant centres were split and tested for the two tests side by side. Xpert MTB/RIF Ultra was performed on 1.0 mL from pooled S1 and S2 raw samples. For each pooled sample, 1.0 mL was placed in a tube, and a sample reagent was added (2:1 ratio). The mixture was shaken and incubated for 10 minutes, vortexed, and incubated for another 5 min. If not fully liquefied, samples were shaken again and left at room temperature for another 5–10 min. From the liquefied samples, 2 mL was used in the Xpert MTB/RIF Ultra cartridge.

Truenat MTB Plus tests were performed on pooled S1 and S2 raw samples and on sample S4. For each test, 0.5 mL of sputum was transferred to a tube, and 2 drops of lysis buffer were added. The tube was vortexed for 10 sec, incubated for 10 min, and transferred to a container with an extraction buffer, vortexed, and incubated for another 10 min. The sample was transferred to a DNA extraction cartridge and inserted into the Trueprep AUTO v2 equipment. Finally, 150 μL of extracted DNA was collected, and 6 μL was added to a PCR mix and transferred to a microchip for MTB detection. The microchip was inserted into the Truelab Duo for DNA amplification. If positive, the DNA extract was transferred to the Truenat MTB-RIF Dx chip for the detection of RIF resistance.

Ziehl Neelsen staining for acid-fast bacilli (AFB) was done on the two sputum samples collected on the first day. On Day 2, sample S3 was decontaminated for culture and sample S4 for Truenat. Culture and phenotypic DST on pooled decontaminated samples (S1 and S2) and S3 by MGIT were done. After decontamination by N-acetyl-l-cysteine–sodium hydroxide, the sample was resuspended in 2 mL of phosphate buffer solution, and 0.5 mL of this sample was inoculated into the MGIT tube for growth analysis. After growth was detected by the MGIT equipment, the positive tube was removed, and an aliquot of the grown material was used to confirm the MTB complex by the MPT64 test and verify growth for other bacteria by blood agar. DST included testing for streptomycin (SM), isoniazid (INH), RIF and ethambutol (EMB).

We used a modified version of the original FIND protocol:^20^ 1) we did not perform Xpert MTB/RIF Ultra alongside the decontaminated S2 since all the procedures were done in the reference laboratory and not in a primary health unit laboratory; 2) we did not exclude from the analyses participants who did not provide sample 4 (S4).

### Statistical analysis

Sociodemographic and clinical data were collected at recruitment, including HIV status, TB history, and smear status. To evaluate the accuracy of Truenat MTB Plus in detecting TB and RIF-resistant TB, we calculated sensitivity, specificity, and positive and negative predictive values stratified by HIV status and previous TB history. Five test results were included in the analysis: 1) Xpert MTB/RIF Ultra: results from raw samples S1 and S2; 2) Truenat MTB Plus raw Day 1: results from the direct pool of raw S1 and S2 samples; 3) Truenat MTB Plus Decontaminated Day 1: results from decontaminated S1 and S2 pooled samples; 4) Truenat MTB Plus raw Day 2: results from raw S4 sample; 5) Truenat MTB Plus 1+2: combined result from Truenat MTB Plus raw Day 1 and raw Day 2. Truenat MTB Plus results were interpreted as follows: if Truenat MTB Plus 1 or Truenat MTB Plus 2 was positive, Truenat result was considered positive; if both were negative, Truenat result was negative. Raw samples from Day 1 (S1 and S2) were used to compare the accuracy between Xpert MTB/RIF Ultra and Truenat MTB Plus.

Agreement between Truenat MTB Plus and Xpert MTB/RIF Ultra was assessed using the kappa test. Sensitivity, specificity, and 95% confidence intervals (CIs) were calculated for each test. Statistical analysis was performed using R software (R Computing, Vienna, Austria).

### Ethical issues

The study followed the Declaration of Helsinki and Brazilian Resolution 466/12. It was approved by the Research Ethics Committees, including the National Research Ethics Committee (CAAE 13121519.3.1001.5257). Participation was voluntary, with written informed consent from all participants.

## RESULTS

### Descriptive analysis

**Figure 1.**
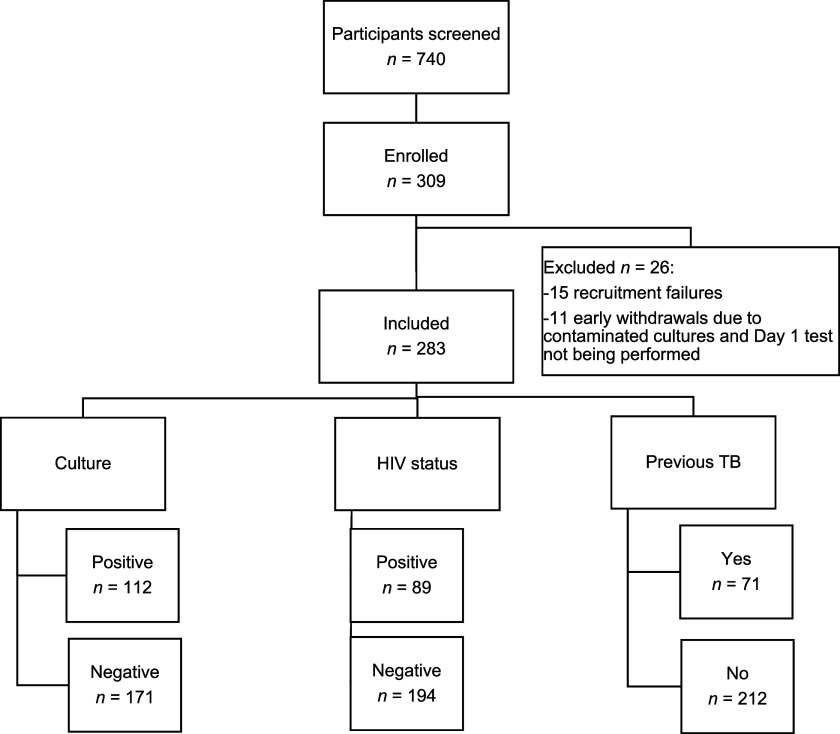
Flowchart of the study population.

Between July 2021 and December 2023, 740 patients with pPTB were screened, and 309 were enrolled across the four study sites (Figure 1). Of these, 112 (39.6%) had positive cultures for MTB, with 47.3% of them AFB-negative. Xpert MTB/RIF Ultra revealed RIF resistance in 11 of 88 samples (12.5%), while Truenat MTB Plus detected resistance in 9 of 72 samples (12.5%). The sociodemographic and clinical characteristics of participants are described in Table 1.

**Table 1.** Demographic and clinical characteristics of participants stratified by TB status.

		TB	Non-TB	
	Total	(*n* = 112)	(*n* = 171)	
	*n* (%)	*n* (%)	*n* (%)	*P*-value
Age, years, median [IQR]	43.0 [32.0–55.0]	39.0 [28.0–54.2]	45.0 [34.0–56.5]	0.025
Age group, years				0.091
18–24	25 (8.8)	14 (12.5)	11 (6.4)	
25–59	205 (72.4)	82 (73.2)	123 (71.9)	
60 years or older	53 (18.7)	16 (14.3)	37 (21.6)	
Sex				0.4
Female	99 (35.0)	36 (32.1)	63 (36.8)	
Male	184 (65.0)	76 (67.9)	108 (63.2)	
BMI				0.003
Underweight	62 (25.9)	33 (31.7)	29 (21.5)	
Normal weight	122 (51.0)	59 (56.7)	63 (46.7)	
Overweight	34 (14.2)	8 (7.7)	26 (19.3)	
Obesity	21 (8.8)	4 (3.8)	17 (12.6)	
Missing	44	8	36	
Clinical status				0.2
Healthy	36 (12.8)	16 (14.3)	20 (11.8)	
Slightly sick	170 (60.3)	66 (58.9)	104 (61.2)	
Moderately ill	66 (23.4)	29 (25.9)	37 (21.8)	
Seriously ill	10 (3.5)	1 (0.9)	9 (5.3)	
Missing	1	0	1	
HIV	89 (31.4)	20 (17.9)	69 (40.4)	<0.001
Previous TB	71 (25.1)	29 (25.9)	42 (24.6)	0.8
Cough				0.5
Negative	2 (0.7)	0 (0.0)	2 (1.2)	
Positive	281 (99.3)	112 (100.0)	169 (98.8)	
Fever				0.047
Negative	90 (31.8)	28 (25.0)	62 (36.3)	
Positive	193 (68.2)	84 (75.0)	109 (63.7)	
Sweat				0.015
Negative	100 (35.3)	30 (26.8)	70 (40.9)	
Positive	183 (64.7)	82 (73.2)	101 (59.1)	
Weight loss				0.006
Negative	70 (25.0)	18 (16.2)	52 (30.8)	
Positive	210 (75.0)	93 (83.8)	117 (69.2)	
Missing	3	1	2	
Haemoptysis				0.4
Negative	202 (71.4)	77 (68.8)	125 (73.1)	
Positive	81 (28.6)	35 (31.2)	46 (26.9)	
Other symptoms				0.091
Negative	66 (23.3)	32 (28.6)	34 (19.9)	
Positive	217 (76.7)	80 (71.4)	137 (80.1)	
MGIT culture Day 1				<0.001
Negative	174 (63.5)	10 (9.1)	164 (100.0)	
Positive	100 (36.5)	100 (90.9)	0 (0.0)	
Missing	9	2	7	
MGIT culture Day 2				<0.001
Negative	156 (63.7)	12 (11.9)	144 (100.0)	
Positive	89 (36.3)	89 (88.1)	0 (0.0)	
Missing	38	11	27	
AFB-indeterminate				<0.001
Negative	224 (79.2)	53 (47.3)	171 (100.0)	
Positive	59 (20.8)	59 (52.7)	0 (0.0)	
Xpert MTB/RIF Ultra				<0.001
Negative	188 (66.4)	24 (21.4)	164 (95.9)	
Positive	88 (31.1)	87 (77.7)	1 (0.6)	
Inconclusive	7 (2.5)	1 (0.9)	6 (3.5)	
Truenat raw Day1				<0.001
Negative	196 (70.0)	30 (27.0)	166 (98.2)	
Positive	81 (28.9)	80 (72.1)	1 (0.6)	
Indeterminate	3 (1.1)	1 (0.9)	2 (1.2)	
Missing	3	1	2	
Truenat decontaminated Day1				<0.001
Negative	191 (68.2)	29 (25.9)	162 (96.4)	
Positive	89 (31.8)	83 (74.1)	6 (3.6)	
Missing	3	0	3	
Truenat raw Day 2				<0.001
Negative	177 (68.1)	33 (31.1)	144 (93.5)	
Positive	77 (29.6)	71 (67.0)	6 (3.9)	
Indeterminate	6 (2.3)	2 (1.9)	4 (2.6)	
Missing	23	6	17	
Truenat RIF-resistant				>0.9
Resistant	9 (12.3)	9 (12.5)	0 (0.0)	
Sensitive	64 (87.7)	63 (87.5)	1 (100.0)	
Missing	210	40	170	
Xpert RIF-resistant				>0.9
Resistant	12 (12.6)	11 (12.5)	1 (14.3)	
Sensitive	83 (87.4)	77 (87.5)	6 (85.7)	
Missing	188	24	164	

IQR = interquartile range; BMI = body mass index; MGIT = Mycobacteria Growth Indicator Tube; AFB = acid-fast bacilli; RIF = rifampicin.

Sputum smear positivity was lower in PLHIV than in HIV-negative individuals (20% vs 59.8%; *P* = 0.001), and MTB detection rates in Xpert MTB/RIF Ultra were lower in PLHIV (50% vs 83.7%; *P* = 0.001). Truenat MTB Plus also showed lower positivity in PLHIV, particularly in decontaminated samples (40% vs 81.5%; *P* < 0.001) (Table 2).

**Table 2.** Laboratory results stratified by HIV status among participants classified as TB or non-TB.

HIV	TB				Non-TB			
		HIV-negative	HIV-positive			HIV-negative	HIV-positive	
	Total	(*n* = 92)	(*n* = 20)		Total	(*n* = 102)	(*n* = 69)	
	*n* (%)	*n* (%)	*n* (%)	*P*-value	*n* (%)	*n* (%)	*n* (%)	*P*-value
AFB				0.001				>0.9
Negative	53 (47.3)	37 (40.2)	16 (80.0)		171 (100.0)	102 (100.0)	69 (100.0)	
Positive	59 (52.7)	55 (59.8)	4 (20.0)		0 (0.0)	0 (0.0)	0 (0.0)	
Xpert Ultra				0.001				0.4
Negative	24 (21.4)	15 (16.3)	9 (45.0)		164 (95.9)	96 (94.1)	68 (98.6)	
Positive	87 (77.7)	77 (83.7)	10 (50.0)		1 (0.6)	1 (1.0)	0 (0.0)	
Inconclusive	1 (0.9)	0 (0.0)	1 (5.0)		6 (3.5)	5 (4.9)	1 (1.4)	
Truenat raw Day 1				0.002				>0.9
Negative	30 (27.3)	19 (21.1)	11 (55.0)		166 (99.4)	99 (99.0)	67 (100.0)	
Positive	80 (72.7)	71 (78.9)	9 (45.0)		1 (0.6)	1 (1.0)	0 (0.0)	
Indeterminate	1 (0.9)	1 (1.1)	0 (0.0)		2 (1.2)	2 (2.0)	0 (0.0)	
Missing	1	1	0		2	0	2	
Truenat decontaminated Day 1				<0.001				0.7
Negative	29 (25.9)	17 (18.5)	12 (60.0)		162 (96.4)	98 (97.0)	64 (95.5)	
Positive	83 (74.1)	75 (81.5)	8 (40.0)		6 (3.6)	3 (3.0)	3 (4.5)	
Missing					3	1	2	
Truenat raw Day 2				<0.001				>0.9
Negative	33 (31.7)	20 (23.5)	13 (68.4)		144 (96.0)	83 (95.4)	61 (96.8)	
Positive	71 (68.3)	65 (76.5)	6 (31.6)		6 (4.0)	4 (4.6)	2 (3.2)	
Indeterminate	2 (1.9)	2 (2.6)	0 (0.0)		4 (2.6)	4 (4.4)	0 (0.0)	
Missing	6	5	1		17	11	6	
MGIT SIRE				0.2				
Resistant	20	17	3					
MDR	9 (12.2)	8 (12.1)	1 (12.5)					
EMB	5 (6.8)	5 (7.6)	0 (0.0)					
SM	2 (2.7)	1 (1.5)	1 (12.5)					
INH	4 (5.4)	3 (4.5)	1 (12.5)					
Susceptible	54 (73.0)	49 (74.2)	5 (62.5)					
Missing	38	26	12					

AFB = acid-fast bacilli; MGIT = Mycobacteria Growth Indicator Tube; MDR = multidrug-resistant; EMB = ethambutol; SM = streptomycin; INH = isoniazid.

AFB positivity was lower in participants with previous TB (37.9% vs 57.8%; *P* = 0.065) (Table 3). DST found resistance in 27% (20/74) of samples, with 9 cases of multidrug-resistant TB (MDR-TB), 4 cases of monoresistance to INH, 5 cases of resistance to ethambutol, and 2 to SM.

**Table 3.** Laboratory results stratified by previous TB among participants categorised as TB or non-TB.

	TB				Non-TB			
		No previous TB	Previous TB			No previous TB	Previous TB	
	Total	(*n* = 83)	(*n* = 29)		Total	(*n* = 129)	(*n* = 42)	
	*n* (%)	*n* (%)	*n* (%)	*P*-value	*n* (%)	*n* (%)	*n* (%)	*P*-value
AFB				0.065				>0.9
Negative	53 (47.3)	35 (42.2)	18 (62.1)		171 (100.0)	129 (100.0)	42 (100.0)	
Positive	59 (52.7)	48 (57.8)	11 (37.9)		0 (0.0)	0 (0.0)	0 (0.0)	
Xpert Ultra				0.7				0.2
Negative	24 (21.4)	19 (22.9)	5 (17.2)		164 (95.9)	125 (96.9)	39 (92.9)	
Positive	87 (77.7)	63 (75.9)	24 (82.8)		1 (0.6)	0 (0.0)	1 (2.4)	
Inconclusive	1 (0.9)	1 (1.2)	0 (0.0)		6 (3.5)	4 (3.1)	2 (4.8)	
Truenat raw Day1				0.4				0.2
Negative	30 (27.3)	24 (29.6)	6 (20.7)		166 (99.4)	127 (100.0)	39 (97.5)	
Positive	80 (72.7)	57 (70.4)	23 (79.3)		1 (0.6)	0 (0.0)	1 (2.5)	
Indeterminate	1 (0.9)	1 (1,2)	0 (0.0)		2 (1.2)	2 (1.6)	0 (0.0)	
Missing	1	1	0		2	0	2	
Truenat decontaminated Day 1				0.8				0.6
Negative	29 (25.9)	22 (26.5)	7 (24.1)		162 (96.4)	122 (96.8)	40 (95.2)	
Positive	83 (74.1)	61 (73.5)	22 (75.9)		6 (3.6)	4 (3.2)	2 (4.8)	
Missing					3	3	0	
Truenat raw Day 2				0.7				0.6
Negative	33 (31.7)	25 (32.9)	8 (28.6)		144 (96.0)	109 (96.5)	35 (94.6)	
Positive	71 (68.3)	51 (67.1)	20 (71.4)		6 (4.0)	4 (3.5)	2 (5.4)	
Indeterminate	2 (1.9)	2 (2.6)	0 (0.0)		4 (2.6)	3 (2.6)	1 (2.6)	
Missing	6	5	1		17	13	5	
MGIT SIRE				0.8				
Resistant	20	14	6					
MDR	9 (12.2)	5 (9.4)	4 (19.0)					
EMB	5 (6.8)	4 (7.5)	1 (4.8)					
SM	2 (2.7)	2 (3.8)	0 (0.0)					
INH	4 (5.4)	3 (5.7)	1 (4.8)					
Susceptible	54 (73.0)	39 (73.6)	15 (71.4)					
Missing	38	30	8					

AFB = acid-fast bacilli; MGIT = Mycobacteria Growth Indicator Tube; MDR = multidrug-resistant; EMB = ethambutol; SM = streptomycin; INH = isoniazid.

### Diagnostic accuracy of Truenat MTB Plus and Xpert MTB/RIF Ultra

Using pooled raw Day 1 and Day 2 respiratory specimens, Truenat MTB Plus showed an overall sensitivity of 72.7% (95% CI 63.41–80.78) and specificity of 99.4% (95% CI 96.71–99.98). The analysis using raw Day 1 and decontaminated Day 1 respiratory specimens presented a lower sensitivity and a higher specificity than pooled raw Day 1 and Day 2 respiratory specimens. Xpert MTB/RIF Ultra had a sensitivity of 78.4% (95% CI 69.56–85.63) and a specificity of 99.4% (95% CI 96.67–99.98) in Day 1 raw samples.

Among 89 PLHIV, Truenat MTB Plus raw 1 + 2 had a sensitivity of 45.0% (95% CI 23.06–68.47) and specificity of 97.1% (95% CI 89.78–99.64), while Xpert MTB/RIF Ultra had a sensitivity of 52.6% (95% CI 28.86–75.55) and specificity of 100% (95% CI 94.72–100). For 194 HIV-negative patients, Truenat MTB Plus raw Day 1 sensitivity was 78.9% (95% CI 69.01–86.79) and specificity 99.0% (95% CI 94.55–99.97), while Xpert MTB/RIF Ultra showed a sensitivity of 83.7% (95% CI74.54–90.58) and specificity of 99.0% (95% CI 94.39–99.97). Truenat raw Day 1 presented a similar sensitivity to Truenat Day 1 + 2. Truenat decontaminated on Day 1 and Truenat on Day 2 had lower values in the PLHIV group. All tests showed a specificity higher than 95% in PLHIV and HIV-negative patients.

**Figure 2.**
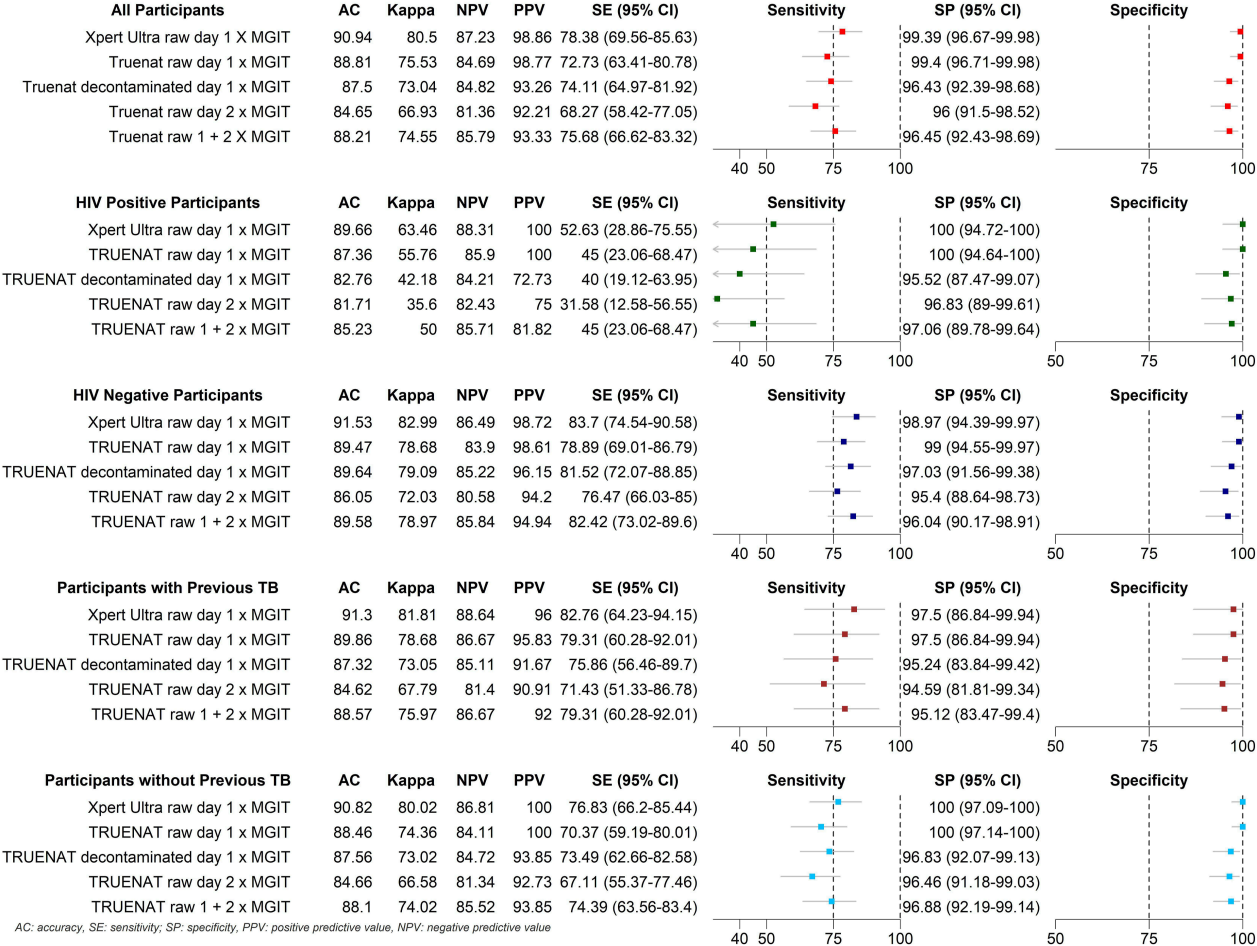
Comparative sensitivity of Truenat MTB Plus tests across specimen types and days stratified by HIV status and history of previous TB treatment. AC = accuracy; SE = standard error; NPV = negative predictive value; PPV = positive predictive value; CI = confidence interval; MGIT = Mycobacteria Growth Indicator Tube.

In patients with a history of previous TB (*n* = 71), Truenat MTB Plus raw Day 1 had a sensitivity of 79.3% (95% CI 60.28–92.01) and specificity of 97.5% (95% CI 86.84–99.94). Xpert MTB/RIF Ultra showed a sensitivity of 82.8% (95% CI 64.23–94.15) and a specificity of 97.5% (95% CI 86.84–99.94). Sensitivity results for individual Truenat MTB Plus tests (raw 1+2, decontaminated Day 1, and raw Day 2) were equal to or lower across all groups (Figure 2). For smear-negative participants, Truenat MTB Plus and Xpert MTB/RIF Ultra sensitivities were respectively 42.3% (95% C 28.73–56.80) and 53.9% (95% CI 39.47–67.77) with no significant variation by HIV status (data not shown).

Kappa test results showed no significant performance difference between Truenat MTB Plus and Xpert MTB/RIF Ultra compared to MGIT in HIV-negative patients with TB, those with TB history, or HIV status. The agreement between Truenat MTB Plus and Xpert MTB/RIF Ultra was 0.9573. Indeterminate results occurred in 3.2% (9/283) of Truenat MTB Plus tests, compared to 2.5% (7/283) for Xpert MTB/RIF Ultra.

## DISCUSSION

Despite the WHO recommending Truenat MTB Plus for TB diagnosis in 2020, few studies have compared its accuracy to Xpert MTB/RIF Ultra.^11,18,21^ In our study, the first in Brazil, the accuracy of Truenat MTB Plus was slightly lower but comparable to Xpert MTB/RIF Ultra with high agreement between the tests, consistent with the findings of Gomathi et al.,^14^ where similar sensitivities were described (76% for Truenat MTB Plus and 78% for Xpert MTB/RIF). In the multicentric study conducted by Penn-Nicholson et al.,^11^ only in Peru was the Truenat MTB Plus compared to Xpert MTB/RIF Ultra instead of Xpert MTB/RIF. In this country, the authors found a higher sensitivity of Xpert MTB/RIF Ultra (95%) compared to Truenat MTB Plus (79%) when evaluating a sample size of patients with pulmonary TB close to ours (378 patients tested). In Uganda and Cameroon, sensitivities were high for Truenat MTB Plus (80.5% and 91%, respectively) and for Xpert MTB/RIF Ultra (96%; available only in the Cameroon study).^18,21^ No significant differences in specificity between Truenat MTB Plus and Xpert MTB/RIF Ultra were observed, consistent with findings from other studies.^11,18,21^

In most studies comparing Truenat MTB Plus and Xpert MTB/RIF, the sensitivities in patients with HIV infection and in those who had previous TB were not reported. Overall, no significant differences in sensitivity and specificity were observed in the studies that compared the accuracy of these two tests.^11,12,14,16,21^ Few studies found higher sensitivity with Truenat MTB Plus, improving TB detection in these populations.^11,21^ Additionally, no differences in sensitivity of Truenat MTB Plus and Xpert MTB/RIF Ultra were noted for smear-negative samples,^11^ which aligns with our findings.

Among PLHIV included in our study, the sensitivity of Truenat MTB Plus was similar to Xpert MTB/RIF Ultra (45% vs 53%), consistent with findings from Penn-Nicholson et al. in the multicenter study involving 48 PLHIV and Ngangue et al. in Cameroon with 74 PLHIV.^11,21^ The specificities of Truenat MTB Plus and Xpert MTB/RIF Ultra (97.5%) in patients with a previous history of TB were also similar, differing from the report on Xpert MTB/RIF reviewed by the WHO in 2020.^2^

RIF resistance detection in our study (12.5%) was slightly lower than described by Penn Nicholson et al. (15%).^11^ Both Truenat MTB Plus (3.2%) and Xpert MTB/RIF Ultra (2.5%) had low rates of indeterminate results, similar to findings described by Jeyashree et al.,^12^ that found 3.6% of indeterminate results for Truenat MTB Plus. Two cases of resistance to RIF were detected by Xpert MTB/RIF Ultra, but not by Truenat MTB Plus, maybe due to the differences in accuracy in detecting RIF resistance between the two molecular tests. Additional information on the *rpo*B mutation profile from other assays for these two cases was not further explored since it was not part of the protocol.

Strengths of our study include the rigorous methodology, adherence to the FIND protocol used by Penn Nicholson et al.,^11^ use of a robust reference standard, and a direct comparison of Truenat MTB Plus with Xpert MTB/RIF Ultra. Our sample included 31% PLHIV, making the results more applicable to high HIV-TB burden settings.

Study limitations include the small sample size of some sub-groups (patients with negative smear and previous TB), the lack of evaluation of Truenat MTB Plus in primary health unit laboratories, and no user satisfaction assessment (laboratory technician and TB manager), as recommended by Engel et al.^22^

## CONCLUSION

Although Xpert MTB/RIF Ultra showed slightly higher accuracy, Truenat MTB Plus demonstrated a diagnostic performance very similar to Ultra, offering a valuable option for TB diagnosis in diverse populations, including PLHIV, patients with a history of TB in the past, and individuals with AFB-negative results. The availability of rapid results for TB, the minimal infrastructure requirements, and the high performance make Truenat MTB Plus a practical alternative to Xpert MTB/RIF Ultra in Brazil's Unified Health System, particularly in primary healthcare settings where initial care for patients with presumptive TB occurs.
